# Identifying symptom etiologies using syntactic patterns and large language models

**DOI:** 10.1038/s41598-024-65645-6

**Published:** 2024-07-13

**Authors:** Hillel Taub-Tabib, Yosi Shamay, Micah Shlain, Menny Pinhasov, Mark Polak, Aryeh Tiktinsky, Sigal Rahamimov, Dan Bareket, Ben Eyal, Moriya Kassis, Yoav Goldberg, Tal Kaminski Rosenberg, Simon Vulfsons, Maayan Ben Sasson

**Affiliations:** 1 Allen Institute for AI, Seattle, USA; 2 Faculty of Biomedical Engineering, Technion, Haifa, Israel; 3 Computer Science Department, Bar Ilan University, Ramat Gan, Israel; 4 Technion Faculty of Medicine Library and Rambam Health Campus Library, Haifa, Israel; 5 Institute for Pain Medicine, Rambam Health Campus, Haifa, Israel; 6 Alan Edwards Pain Management Unit, McGill University Health Centre, Montreal, QC Canada

**Keywords:** Diseases, Signs and symptoms, Computer science, Scientific data, Information technology

## Abstract

Differential diagnosis is a crucial aspect of medical practice, as it guides clinicians to accurate diagnoses and effective treatment plans. Traditional resources, such as medical books and services like UpToDate, are constrained by manual curation, potentially missing out on novel or less common findings. This paper introduces and analyzes two novel methods to mine etiologies from scientific literature. The first method employs a traditional Natural Language Processing (NLP) approach based on syntactic patterns. By using a novel application of human-guided pattern bootstrapping patterns are derived quickly, and symptom etiologies are extracted with significant coverage. The second method utilizes generative models, specifically GPT-4, coupled with a fact verification pipeline, marking a pioneering application of generative techniques in etiology extraction. Analyzing this second method shows that while it is highly precise, it offers lesser coverage compared to the syntactic approach. Importantly, combining both methodologies yields synergistic outcomes, enhancing the depth and reliability of etiology mining.

## Introduction

Differential diagnosis is a cornerstone of modern medicine, holding paramount importance in the field. It serves as a systematic and meticulous process that allows healthcare professionals to distinguish between various potential medical conditions with similar symptoms. By considering a range of possible diagnoses and eliminating them one by one, clinicians can arrive at an accurate diagnosis, ensuring that patients receive appropriate treatment and care. This critical step not only enhances patient outcomes but also minimizes the risks of misdiagnosis and unnecessary procedures, highlighting the indispensable role of differential diagnosis in the practice of medicine.

Clinicians often consult resources such as books, review papers, or summarization services like UpToDate^1^ in order to understand possible etiologies for a set of symptoms. However, these resources may be limited in their ability to incorporate the latest findings or less common ones, as they are manually curated. Automated text mining has been proposed as a solution to address these limitations, but text mining efforts have typically focused on small subsets of data or required NLP (Natural Language Processing) experts to operate complex pipelines^2–7^. As a result, these efforts have not led to the creation of up-to-date resources widely used by the scientific community.

In this study, we suggest two methods for mining etiologies from the scientific literature. The first method is rooted in traditional NLP and makes use of syntactic patterns and iterative bootstrapping. In contrast to previous studies in pattern bootstrapping which relied on fully automated pattern creation^2,8,9^, we advocate a human-in-the-loop approach. We developed a dedicated bootstrapping user interface (UI) and showed that by using it, a set of syntactic patterns could be obtained with a reasonable human effort (4 h) and that the patterns could be used to reliably extract symptom etiologies with high coverage.

The second method makes use of the ChatGPT-4 (Chat-Generative Pre-trained Transformer-4) generative model to generate etiologies and employs a fact verification pipeline to remove hallucinations and provide reliable provenance information. This is to our knowledge the first systematic application of generative methods to etiology extraction. We showed that this method can be highly accurate, but has lower coverage compared to syntactic patterns running on a large corpus. This is consistent with recent findings about the difficulty of LLMs (Large Language Models) in encoding the long tail of world knowledge^10,11^. Importantly, as we’ll show in the “Results” section, the method of syntactic patterns and the generative method complement each other, and the best overall results are obtained by combining both.

**Figure 1:**
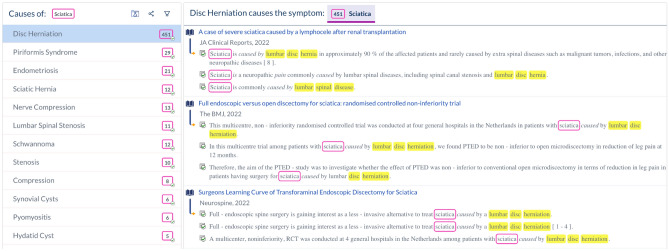
A screenshot of a designated web interface, allowing researchers and clinicians to query for symptom etiologies (https://cq.allen.ai).

An important feature of this study is that the relevant extraction patterns and GPT (Chat-Generative Pre-trained Transformer) prompts may be applied to extract symptom etiologies from live and up-to-date resources, at interactive speed. While the process is described in detail in this paper, we also provide a webpage which abstracts the details and allows researchers and clinicians to search for symptom etiologies using these methods and based on symptom name alone. Figure 1 shows a screenshot of this page, where etiologies for sciatica are listed, along with their provenance information.

The contributions of this study can be summarized as follows.

1. A workflow and dedicated UI to bootstrap syntactic patterns. The workflow has been applied in this study to identify patterns for etiologies, but could easily be applied to other relations between entities.
2. A novel application of ChatGPT with a dedicated fact verification pipeline to the problem of identifying etiologies.
3. A dedicated UI which allows to easily retrieve etiologies from a live and routinely updated snapshot of the scientific literature.
4. Analysis of the current state of text mining for etiology detection, and a comparison to alternative resources such as books, review papers and UpToDate).

## Related work

There has been a significant amount of research on extracting information from the scientific literature, particularly in the biomedical domain^12,13^. Many approaches have focused on extracting common biomedical entities like genes, diseases and drugs and the relationships between them. However, less work has been directed at extracting symptom etiologies.

Extracting etiologies is a critical task for commercial symptom checkers such as WebMD^14^, patient.info^15^ and Isabel^16^ which apply computation inference on top of pre-curated symptom-disease data. The data required for these systems is acquired from medical books and top journals and is curated manually by medical experts^17^. The data is accurate but requires domain expertise and vast amounts of time to curate and update. Furthermore, rare or very recently discovered etiologies are often overlooked and the curated etiologies lack fine-grained frequency information.

An alternative approach for disease-symptom extraction is based on data mining and co-occurrence. This involves extracting diseases and symptoms from medical sources, normalizing the extracted names based on medical vocabularies (relevant vocabularies are DO, SYMP, UMLS, MeSH, MEDIC, SNOMED-CT) and using disease-symptom co-occurrence (e.g. co-occurrence in the same PubMed abstract, or the same EMR report) to statistically model the disease-symptom connections. This approach is typically used in the construction of disease networks, where the extracted disease-symptoms associations are used to populate a knowledge graph, often along with other types of connections like disease-gene or disease protein^7,18–20^. Disease networks include fine-grained frequency information and are useful for statistical inference, discovering connections between seemingly unrelated diseases and for drug repurposing. However, this approach does not directly extract causal disease-symptom relations, and the reliance on co-occurrence introduces a degree of noise which makes it less relevant for a clinical setting where clinicians require etiologies to be accurate and/or easily validated by accompanying evidence.

More similar to our approach are studies using syntactic patterns over dependency trees to extract disease-symptom relations^2,4^. For example, Hassan et al.^4^ manually annotated a dataset of labelled relations (PubMed sentences with at least diseases and symptom spans, and a set of zero or more binary relations between disease-symptom pairs) and used it to generate syntactic patterns. The generated patterns were then ranked and selected based on quality criteria such as the ratio of the correct pattern extractions to the total extractions. Our work differs from these approaches by not relying on labelled data and instead employing iterative bootstrapping and a dedicated UI facilitating a human-in-the-loop approach to pattern creation (see section “Results”/“Syntactic patterns and iterative bootstrapping”). In addition, these earlier works did not release models, training data or extractions, so their outputs are not available to medical professionals.

Concerning Machine Learning (ML) modelling, Disease Named Entity Recognition (NER) datasets have existed for a while^21,22^, but a dataset for symptom extraction was only recently released^23^. Furthermore, Feng et al.^3^ are as far as we know the only ones applying ML techniques to disease-symptom extraction. This is most likely due to the absence of sizable training data for this problem. In their study Feng et al. trained kernel support vector machines (SVMs) for relation disease-symptom relation classification in a semi-supervised setting. (In part of this work they did hand label a small ad-hoc dataset to train and evaluate their model, but their main contribution was at developing models that can utilize unlabeled data). Models, code or extractions were not made available as part of this work.

## Results

Below we briefly describe the suggested methods for etiology extraction and evaluate their results.

### Syntactic patterns and iterative bootstrapping

**Figure 2:**
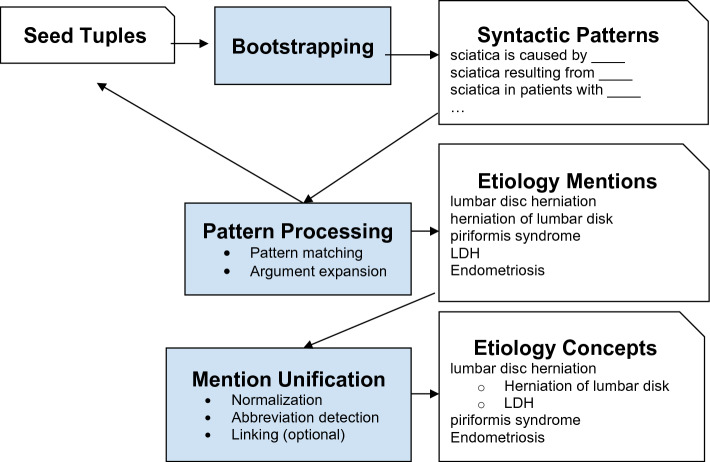
Extracting etiologies with syntactic patterns and iterative bootstrapping.

Figure 2 shows the general architecture of our approach, comprising three main stages: (1) bootstrapping: a dedicated UI component allowing pattern developers to quickly identify syntactic extraction templates based on a handful of result examples. The UI component has been made publicly available as part of the SPIKE extractive search system. The SPIKE system is a free online information extraction platform available at https://spike.apps.allenai.org. As part of this work we have added to it a module for bootstrapping syntactic patterns which is described in the “Methods” section; (2) extraction: the system applies the patterns, removes duplicates from the results and obtains sentences with etiology mentions; and (3) mention unification: the extracted mentions are unified into groups of synonymous mentions (“concepts”). The concepts are then ranked by their number of mentions. In the methods section we describe each of these stages in more detail.

### Generating etiologies using ChatGPT

The second method we suggest makes use of generative models, specifically ChatGPT. Since generative models suffer from hallucinations, we employ a novel fact verification pipeline with an evidence ranking component to verify the generated etiologies and provide provenance information.

**Figure 3:**
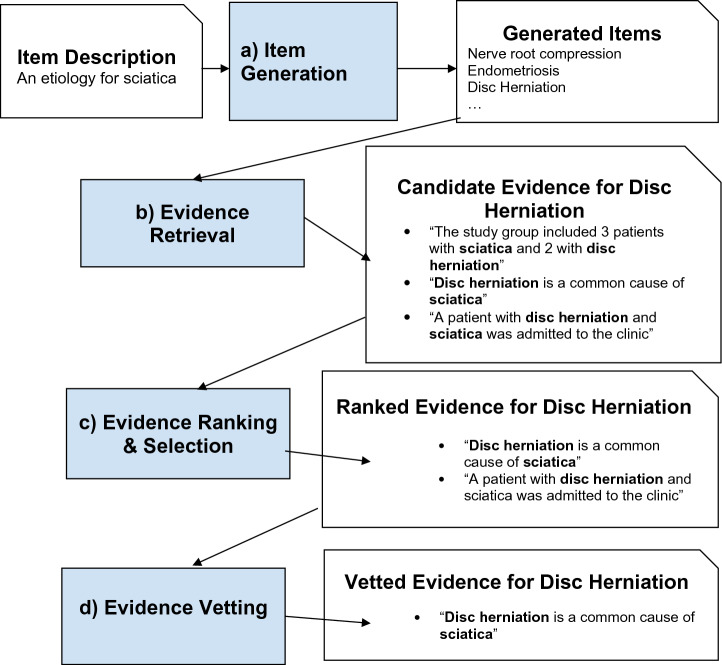
Generating and validating etiologies using ChatGPT and a validation pipeline. The figure shows the verification process for one generated etiology of sciatica, “disc herniation”. In practice, the verification pipeline is applied sequentially to all the generated etiologies, filtering out etiologies without vetted evidence.

Figure 3 describes the process which is comprised of 3 stages: (a) a generative model is prompted to generate an exhaustive list of symptom etiologies; (b) For each generated etiology, we use dense retrieval to obtain N candidate evidence sentences; (c) for each etiology and N candidate evidence sentences, we task a generative model with selecting and ranking n ≪ N sentences which would constitute the best supporting evidence for the etiology. We ask that sentences where the causality is expressed explicitly will be ranked above sentence where it is expressed implicitly, and that sentences that do not express or imply causality between an etiology and the symptom are removed; and (d) we use a final prompt to the language model to verify that the best selected evidence is indeed valid evidence. The “Methods” section describes each of these stages in detail.

### Evaluation

In evaluating the two suggested approaches, we look to measure their recall—the percentage of etiologies they identify out of the full set of etiologies for a symptom—and precision—how many of the etiologies they identify are indeed correct. While estimating the precision of the approaches is possible through sampling, robustly estimating their recall turns out to be challenging since as we’ll see below, reference sources are themselves very incomplete, and so there is no reliable source for the full set etiologies for a symptom. To mitigate this problem, we borrow an evaluation approach used in information retrieval, where etiologies are pooled from several different sources (for each symptom, we use our two suggested approaches to identify etiologies plus a reference source with etiologies known to be correct), results are then manually labelled by experts, and we consider the full set of correctly identified etiologies to be our full set. The recall figure for the different systems is then taken as the percentage of extractions they identify from this full set. This method requires normalizing the extractions of the different systems so that an etiology which appears with different names in the different sources is indeed treated as a single etiology. This method of evaluation is detailed in the section “Comprehensive evaluation with reference sources” and it allows us to estimate the recall of the different approaches and point out their limitations. However, due to the large number of etiologies extracted for each system, and the need for name normalization, this approach does not scale easily to a large number of symptoms.

To further evaluate the precision of our approaches across many symptoms, we randomly select a larger number of symptoms and then several random extractions for each symptom. Annotators then labelled the correctness of these random extractions. The results are detailed in the section “Evaluating precision via random sampling” and provide further evidence for the precision of the two approaches at a larger scale.

### Comprehensive evaluation with reference sources

For this experiment, we extracted etiologies for 3 symptoms: hiccups, jaundice and chest pain. For each of these symptoms, we obtained a list of etiologies from a reference source: for hiccups, we accessed the table “Causes of persistent and intractable hiccups”^24^ in UpToDate on 01/12/2022, for Jaundice we used a book source^25^. For chest pain, we also used an UpToDate summary ^26^ accessed on 01/12/2022.

Since both the syntactic patterns and GPT provide a substantial number of supported etiologies which do not appear in the reference source, we first pool together the etiologies from all sources (the patterns, GPT and the reference), normalize the names manually so that the terminology is the same across sources, and then have a team of trained annotators validate the extractions (we employed a team of 3 annotators, all 4’th year medical students in the Technion medical school, Israel. Annotators were paid for their participation in the annotation effort). Etiologies from the reference source were assumed to be supported by evidence. Etiologies obtained from the Patterns or GPT (and not found in the reference) were labelled as supported/unsupported (correct/incorrect) based on the provided provenance information. both the patterns and the GPT’s verification pipeline provide provenance information in the form of sentences which mention the symptom and the candidate’s etiology and provide information supporting the causal relation between them. The annotators additionally evaluated the correctness of the unvalidated extractions of ChatGPT, in these cases, they were instructed to rely on external sources (namely Google and Pubmed searches) for their annotation.

The annotation effort was supervised by an expert MD (Dr Maayan Ben Sasson). We first measured agreement between the expert and each of the annotators on 20 random extractions (10 from the GPT pool and 10 from the patterns pool) and noted high agreement in all cases. Cohen's kappa agreement was 1 between the expert and the Jaundice annotator, 0.86 between the expert and the Hiccups annotator and 0.77 between the expert and the Chest Pain annotator. Each of the annotators then proceeded to annotate the rest of the extractions for their respective symptoms.

**Figure 4:**
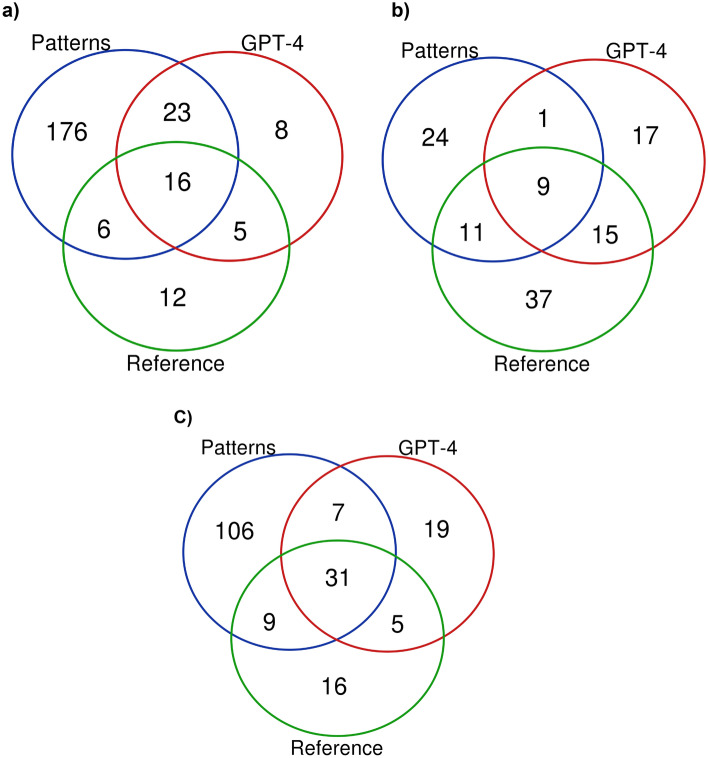
Venn diagrams of correctly identified etiologies by source.

Figure 4 includes Venn diagrams depicting the overlap between the supported etiologies provided by the 3 different sources. As shown in the figure, the patterns have better overall coverage, typically identifying many supported etiologies which are not listed in the reference or identified by ChatGPT. Importantly, despite a degree of overlap, the patterns and GPT have a significant number of supported and non-overlapping extractions, suggesting that it may be useful to combine the two methods.

Table 1 shows the full results for the different systems, taking into account unsupported (incorrect) extractions. The first two rows in Table 1 show the results for the patterns and GPT systems. As expected from the Venn diagrams, the patterns have a substantially higher recall than GPT. This is following recent observations about the difficulty of generative models to handle the “long tail” of corpus facts^10,11^. However, GPT has significantly higher overall precision, even without applying its fact verification pipeline. In the third row of Table 1, we show the results of applying the fact verification pipeline, which brings GPT’s precision to 100% on all symptoms, at the cost of somewhat lower recall.

**Table 1:** Recall, precision and F-scores for the different methods.

	Chest pain			Jaundice			Hiccups			Overall (macro averaged)		
	R	P	F	R	P	F	R	P	F	R	P	F
Patterns	**79.38**	68.44	**73.51**	**85.33**	70.83	**77.41**	**39.82**	86.54	**54.55**	**68.17**	75.27	**68.49**
Chat GPT	31.96	**98.41**	48.25	25.1	**100**	40.12	36.28	85.42	50.93	31.11	94.61	46.43
Chat GPT + verification	25.77	**100**	40.98	18.92	**100**	31.82	25.66	**100**	40.85	23.45	**100**	37.88
Patterns + GPT	**91.75**	**71.2**	**80.18**	**95.37**	**73.07**	**82.75**	**67.26**	84.44	**74.88**	**84.79**	76.24	**79.27**
Patterns + GPTV	88.14	70.66	78.44	93.05	72.59	81.56	56.64	**90.14**	69.57	79.24	**77.8**	76.52

Significant values are in bold.

The final two rows of Table 1 show the results of aggregating the pattern extractions and GPT generations. Unifying the non-verified variant of GPT with the patterns yields the overall highest recall and F-Score compared to all other solutions while unifying the patterns with the verified variant of GPT (labelled GPTV in the table) provides slightly lower Recall and F-Score, but higher precision. Both aggregated variants provide significantly better results than using GPT or the patterns by themselves.

Table 2 shows the recall of the different solution when evaluated only against the reference sources, without considering the full spectrum of verified etiologies. The syntactic patterns still show the highest recall of the non-aggregated methods, and the combination of the patterns and GPT still shows the highest overall recall. However, when evaluating against the reference sources alone, the difference in coverage between the patterns and GPT is much less significant. This is in line with the observation that LLMs are effective at encoding the more common knowledge, which is likely to appear in curated reference sources, and less effective at encoding the long tail knowledge^11^.

**Table 2:** Recall the different methods with respect to the reference source.

	Chest pain	Jaundice	Hiccups	Macro Avg
Syntactic patterns	**65.57**	**56.41**	27.77	**49.92**
Chat GPT	59.02	53.85	**33.33**	48.73
Chat GPT + verification	47.54	48.72	22.22	39.43
Patterns + GPTV	70.49	66.67	40.28	59.15
Patterns + GPT	**73.77**	**69.23**	**48.61**	**63.87**

Significant values are in bold.

Table 3 shows a sample of the extractions of the different systems, the full set of extractions and annotations is available in Supplementary Materials 1.

**Table 3:** A sample of the etiologies identified by the GPT, Patterns and Reference sources. The “Patterns/GPT/Ref” line refers to etiologies found by all 3 sources. The lines: “Patterns”, “GPT” and “Reference” refer respectively to the etiologies identified exclusively by the patterns, GPT or the reference.

Chest pain	Ref/patterns/GPT	Pulmonary hypertension; panic disorders; pericarditis/myopericarditis; eosinophilic esophagitis; fibrositis; pancreatitis; Esophagitis; Esophageal rupture, perforation; pleuritis; myocarditis; tietze syndrome
	Patterns	Manubriosternal joint abnormalities; pleural mesothelioma; mushroom poisoning; thymoma; ADPKD; hyperthyroidism; precordial abscess; esophageal hematoma; chest wall myofibroma; pneumomediastinum
	GPT	Dressler's syndrome; atrial fibrillation; ehlers-danlos syndrome; mediastinitis; lyme disease; ventricular tachycardia; vitamin D deficiency; scorpion sting; angina pectoris; lead poisoning prinzmetal's angina; marfan syndrome
	Reference	Asthma and COPD; systemic lupus erythematosus; xiphoidalgia spontaneous sternoclavicular subluxation; psoriatic arthritis; methamphetamine; sternocostoclavicular hyperostosis (SAPHO syndrome); osteoporosis, osteomalacia
Jaundice	Patterns/GPT/Ref	Choledocholithiasis; lymphoma; primary sclerosing cholangitis; cholangiocarcinoma; extrahepatic biliary atresia; viral hepatitis A, B, D; amyloidosis; tuberculosis; sarcoidosis, strictures, cirrhosis pancreas cancer, dubin-johnson syndrome
	Patterns	Intraorganic liver angiomas; chorioamnionitis; urinary tract infection; stenosing papillitis; intraductal oncocytic papillary neoplasm; abdominal injury; afferent loop syndrome; granulocytic sarcoma; hippel lindau syndrome
	GPT	Crigler-najjar syndrome; crigler-najjar syndrome type II; hereditary spherocytosis; pyruvate kinase deficiency; thalassemia; paroxysmal nocturnal hemoglobinuria
	Reference	Defect of sinusoidal reuptake of conjugated bilirubin; steroids; defect of canalicular organic anion transport; chlorpromazine; jamaican bush tea; HSV; liver flukes; oral contraceptives; rifampin; probenecid; kava kava; arsenic;
Hiccups	Patterns/GPT/Ref	Gastric distention; pleuritis; meningitis; multiple sclerosis; diabetes mellitus; myocardial infarction; intracranial neoplasms; ischemic/hemorrhagic stroke
	Patterns	PICA aneurysm; thoracic aggression; parkinson; right coronary aneurysm; syringobulbia; lateral medullary syndrome; sarcoidosis; renal cyst; metabolic alteration; glaucoma; tetanus; hypertrophic olivary degeneration; brainstem compression; twiddler’s syndrome
	GPT	Ketogenic diet; phrenic nerve irritation; central sleep apnea; diaphragmatic irritation; gastric volvulus; sudden temperature changes; pregnancy; swallowing air; spicy foods; Certain medications (e.g., benzodiazepines, opioids)
	Reference	Hypocapnia; peptic ulcer disease; COVID-19; Intubation (stimulation of glottis); brainstem neoplasms; anorexia nervosa; neurosyphillis; alpha methyldopa; empyema; asthma; short acting barbiturates; hydrocephalus

### Evaluating precision via random sampling

In this experiment, we sampled 20 symptoms from the CTD list of signs and symptoms. (https://ctdbase.org/downloads/;jsessionid=38DDC4627CA9AC7E6EE1120C8284E879#alldiseases.) We then sampled 10 etiologies for each symptom: 5 were sampled randomly from the GPT generations and 5 from the pattern extractions. 2 annotators (An expert MD and a cancer researcher, both authors of this paper) labelled the extractions as 0 (incorrect) and 1 (correct). We measured agreement on 30 extractions before labelling the full set (Cohen’s kappa = 0.71). Table 4 summarizes the results and we include the full annotations in Supplementary Materials 2. The results across all etiologies show 91% precision for ChatGPT and 81% precision for the patterns approach. These results are in line with the precision metrics measured in the comprehensive evaluation of the 3 symptoms (Table 1) where macro-averaged GPT results were slightly higher (94.6%) and the pattern results were slightly lower (75.3%). In the Qualitative Evaluation section, we’ll review some of the mistakes made by both approaches.

**Table 4:** Yield and accuracy of ChatGPT and Patterns approach across an array of symptoms.

	GPT yield	Sample accuracy	Patterns yield	Sample accuracy
Myalgia	62	5/5	430	4/5
Shortness of breath	67	5/5	869	4/5
Anosmia	55	5/5	230	5/5
Photophobia	86	5/5	221	4/5
Impotence	49	5/5	342	5/5
Pelvic pain	54	5/5	303	5/5
Heartburn	27	5/5	106	4/5
Hallucinations	118	5/5	411	4/5
Paresthesia	101	4/5	368	5/5
Confusion	86	5/5	789	3/5
Dysphonia	67	5/5	217	4/5
Hyperhidrosis	46	4/5	103	5/5
Polyuria	76	2/5	214	2/5
Visceral pain	80	5/5	115	4/5
Skin rash	82	5/5	477	4/5
Back pain	66	4/5	646	3/5
Memory deficit	73	5/5	433	4/5
Facial asymmetry	57	5/5	134	3/5
Lymphedema	16	4/5	261	4/5
Anuria	56	3/5	176	5/5
Overall	1324	91%	6845	81%

In Table 4 we also list the yield of the two approaches (number of generated/extracted etiologies for each symptom). While the numbers may be slightly exaggerated due to the lack of manual name normalization, they do further attest to the high number of etiologies identified, especially by the patterns approach.

### Qualitative analysis

To better understand the mistakes made by the two approaches we looked at etiologies missed by each approach (false negatives) and etiologies which were incorrectly identified (false positives). We’ll address the two cases below.

### Missed etiologies

A known limitation of GPT and LLMs is that they can not encode the long tail of rare facts in its parameters^10,11^, so in considering missed etiologies we focused on etiologies listed in our reference sources but not identified by our patterns approach, which has generally high recall. We sampled 20 such etiologies and tracked down the cited sources for these etiologies in the reference. We then mapped out the reasons these sources were not identified by the patterns. The results are summarized in Table 5 below:

**Table 5:** causes for unidentified etiologies, based on 20 random etiologies which appeared in the reference sources but were not identified by the patterns approach.

Reason	Number of cases
Could not validate reference	6
Tabular data, not in abstract	5
Not in abstract	4
Missing pattern	4
Different symptom name	1

The data in the table demonstrates a few limitations of the system. First, for 6 etiologies listed as “Coludn’t validate source,” we were unable to track the relevant cited text. This is either because the cited text is not publicly accessible, or in some cases, the cited text did not mention the etiology. This highlights a limitation of the extractive patterns approach, namely that not all relevant medical information is in the form of digital and publicly accessible publications. This issue cannot be readily addressed, but we’re hopeful that the situation will gradually improve as more publications transition into open-access modes.

Another notable problem which occurred 9 times (the aggregate of “Tabular Data, Not in Abstract” and “Not in Abstract”), is that the relevant information did not appear in the abstracts of PubMed paper, but rather in the body text (either in text form or as tabular data). This should be partially addressed by future updates to the SPIKE system which is expected to cover full texts of ~ 4 million PubMed papers (the PMC collection) in addition to its current coverage of ~ 25 million abstracts. Tabular data is not currently supported in the SPIKE system so this will remain a limitation for the time being and may be addressed by future research.

4 of the missing etiologies were cases where the relevant information appeared in a PubMed abstract, but was not captured by our patterns. A case in point was the “hiccups” symptom, where certain drugs were listed in the reference source as etiologies and our patterns did not identify them. Indeed, patterns for drug side effects were not added as part of our initial bootstrapping effort as these were not relevant for the symptoms considered there. We plan to add additional patterns to address this shortcoming. However, it should be noted that there’s a long tail of ways researchers can indicate causation between an etiology and a symptom, so pattern coverage will always remain incomplete to some degree.

### Incorrectly identified etiologies

To understand the reasons for incorrectly identified etiologies in our two approaches we sampled 10 false positives predicted by each method.

Table 6 summarizes the types of incorrect etiologies generated by GPT. The main source of errors were cases where the generated etiology is related to a correct etiology but is not in itself accurate. Another class of errors are cases where GPT generated correct causes, but these were unlikely to be considered useful etiologies by medical professionals (e.g. due to being too generic and non-specific).

**Table 6:** Incorrect etiologies generated by ChatGPT.

Reason	Count	Example
Generated etiology is close/related to the correct one	5	**Preeclampsia** generated for **clonic seizures**, when the related “eclampsia” would be the correct etiology
The item is too abstract to be a useful etiology	4	**Surgery** generated for **Lymphedema.** Where we sould expect at least the type of surgery to be specified
Incorrect Generation (hallucination)	1	**Hypernatremia** generated as a cause for **polyuria**. We did not find evidence for this

Significant values are in bold.

Table 7 summarizes the types of errors made by the patterns approach. The errors made by pattern application are different from the ones made by GPT and stem from errors made by the different NLP components in the extraction pipeline (e.g. incorrect expansion of a word to its linguistic phrase, parsing errors made by the parser) or by patterns which are not restrictive enough and generate some noise in addition to useful results.

**Table 7:** incorrect etiologies generated by pattern application.

Reason	Count	Example
Incorrect boundaries for etiology	3	**“Polyuria** resulting from cerebral salt **wasting”** The pattern emitted *wasting* as an etiology and not *cerebral salt wasting* as expected
Parsing errors	3	“After exposure to benzoic acid, the following health effects can occur: **eye damage**, …, irritation of the throat if inhaled, which may cause **shortness of breath**” The syntactic parser treated *eye damage* as the subject of the verb *cause*, causing it to be erroneously extracted as an etiology
Pattern indicates relatedness rather than causation	2	“**Dysphonia** associated with **carotidynia** and migraine” The pattern creator has added several patterns which indicate relatedness without clear causation. These patterns can sometimes identify useful etiologies (as in “sciatica associated with disk herniation”) but sometimes extract related symptoms rather than etiologies
Incorrect boundaries for symptom	2	“A presentation of **angioedema** causing **confusion** with child abuse” A pattern extracted angioedema as a cause for the symptom confusion, even though in this context angioedema is causing “confusion with child abuse” and not the symptom confusion

Significant values are in bold.

## Methods

### Extracting etiologies using patterns

#### Extractive search for iterative bootstrapping

We implemented the suggested human-in-the-loop variant of iterative bootstrapping as part of the SPIKE-PubMed extractive search system.

**Figure 5:**
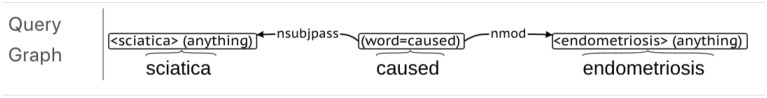
Query graph for the query: <>*:sciatica is $caused by *<>*:endometriosis.*

In extractive search systems^27–29^, users issue queries which consist of a sequence of terms, some of which are designated as capture slots. The capture slots act as variables, whose values should be extracted (“captured”) from matched sentences. For example, the query in (1) is a structured ES query which retrieves all sentences which contain the word “caused”, connected by an edge of *nsubpass* (passive subject) to a word which will be designated as symptom and edge of *nmod* (nominal modifier) to a third word which will be designated as a disease. All PubMed sentences are pre-parsed and indexed and so the syntactic relations between words are being enforced by the matcher. Figure 5 below shows the query graph which corresponds to the structured query.

1. **Structured** <  > :sciatica is $caused by <  > :endometriosis

The dollar preceding the word “caused” indicates that this word should be matched as-is. The colon preceding the terms *sciatica* and *endometriosis* terms indicates that these are captured slots. This means that the matching sentences are not required to include these precise words. Rather they are required to include words that are in a similar syntactic configuration to these terms (i.e. connected by *nsubjpass* and *nmod* edges respectively to the word “caused”, respectively). These captured words are then extracted, expanded to their linguistic phrase boundaries, aggregated and presented to the user as symptom-etiology pairs.

In addition to structured queries that match terms based on their syntactic relationships, extractive search systems provide a “basic” query type, that can match terms irrespective of their position in a sentence as well as other types of queries. The common property of all queries is the ability to designate query terms as capture slots, triggering span extraction in addition to sentence matching.

The support for both syntactic queries and basic/keyword queries lends itself well to implementing a workflow for iterative bootstrapping, as described below in the section “Methods”/“Generating syntactic patterns using iterative bootstrapping”.

#### Generating syntactic patterns using iterative bootstrapping

**Figure 6:**
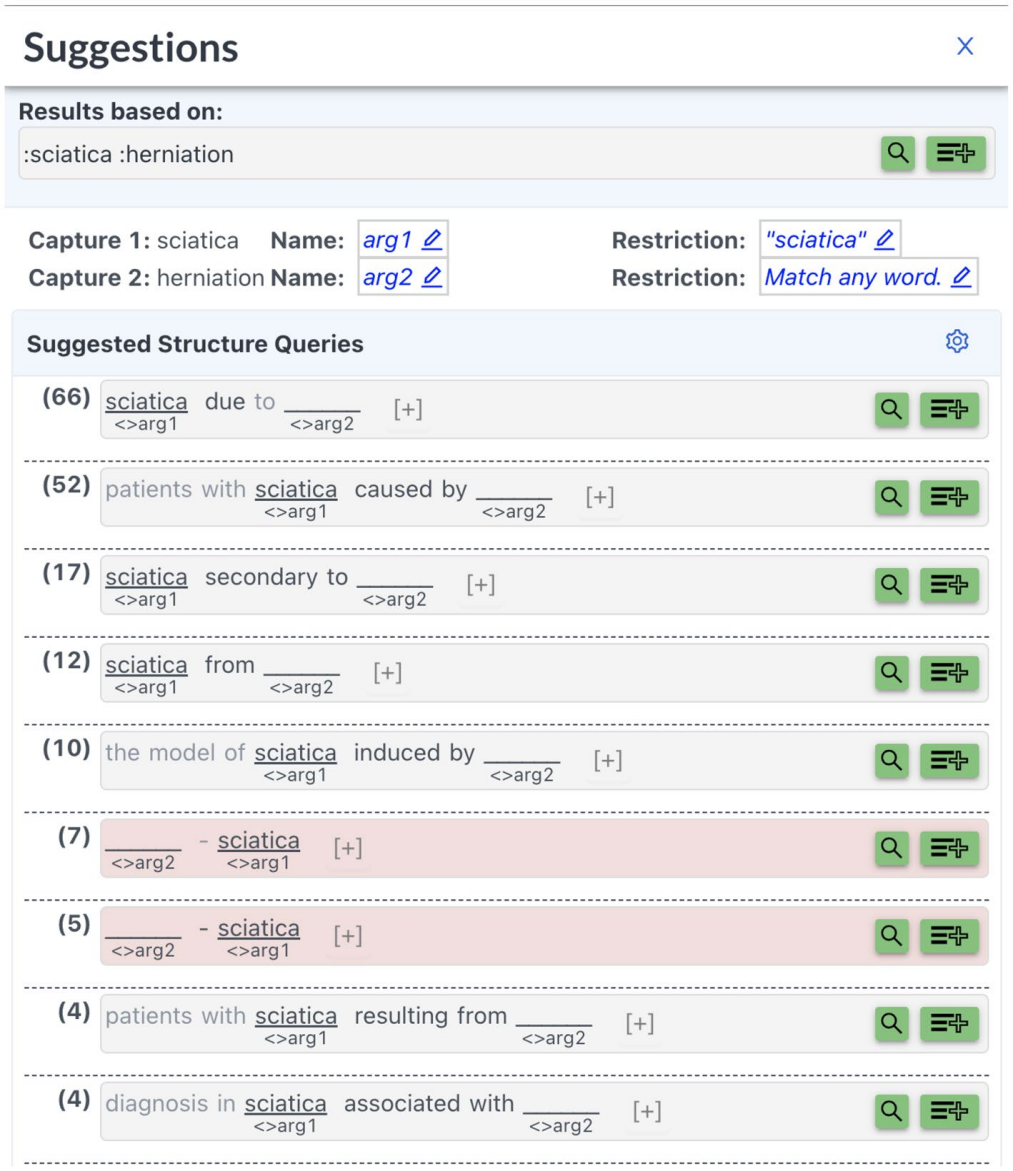
Generating patterns based on the seed tuple <sciatica, herniation>. Note that in practice the generated patterns will not be specific to the word “sciatica”, but would rather be executed for other user-requested symptoms.

As part of this study, we extended the SPIKE-PubMed extractive search system with a module for iterative bootstrapping (it is now integrated into the SPIKE engine as part of its “Suggestions” section). Using this module, pattern developers start by defining one or more seed tuples expressing the relation of interest (e.g. <sciatica, herniation> was the only initial seed tuple used in generating patterns for the symptom-etiology relation). The system then searches PubMed for sentences that contain both tuple elements of one of the seed tuples. The system then automatically generates syntactic patterns from each matching sentence based on the syntactic path between the two matched elements (see “Methods”/“Generating patterns from matching sentences”). Each pattern has a user-friendly text representation and the patterns are ranked by yield. The pattern developer can quickly inspect the yield of each pattern and decide whether to accept or reject it. The system then uses the selected patterns to extract more seed tuples and the process is repeated iteratively. The pattern developer can choose to stop the process once the newly discovered patterns become very specific as indicated by their low yield (e.g. when a pattern matches only the sentence from which it was generated, or up to N additional sentences). Figure 6 shows a screenshot of the initial set of patterns suggested by the bootstrapping component based on the <sciatica, herniation> seed tuple. In parenthesis, before each pattern, we see the number of matched sentences which triggered it. The pattern developer can click the search button (the magnifying glass) to inspect the sentences, and press the plus icon to add a pattern to the pattern pool.

The UI was used by a medical information specialist to derive extraction patterns for the symptom-etiology *caused by* relation in the following manner:

1. Starting from a single seed <sciatica, herniation>, the system retrieved all PubMed sentences which contained the words *herniation* and *sciatica*
2. The system generated patterns from all matching sentences, and the information specialist inspected all patterns with a yield greater or equal to 2. 16 initial patterns were selected and added to the pattern pool. (Note that at this stage, the information specialist quickly inspected the pattern yield, and eliminated patterns with low precision. For example a pattern like “sciatica with _____” was suggested as it’s common to find etiologies in the position of the placeholder (e.g. “sciatica with disk herniation”), however, the pattern was discarded by the information specialist as it does not imply causation and frequently extracts results like “symptoms included sciatica with pain and numbness” where pain and numbness are not etiologies.)
3. The system then ran the selected patterns yielding new symptoms and etiologies. We collected the top 10 symptoms and top 50 etiologies for another round of iterative bootstrapping.
4. We re-ran steps 1–3 with the new seeds, again inspecting patterns with yield greater or equal to 2. This resulted in an additional 44 patterns reaching a total of 60 patterns.

Re-applying the process did not result in new high-yield patterns so we stopped after two iterations. The full set of patterns is given in Supplementary Materials 3.

#### Generating patterns from matching sentences

The input to the pattern generation process is a sentence *S* and a pair of tuple elements in S, denoted with A and B (e.g. the sentence S may be “Sciatica is caused by disk herniation” and the matched tuple elements are A = ” Sciatica” and B = ” disk herniation”). We’ll denote with T the universal (basic) dependency tree for S.

**Figure 7:**
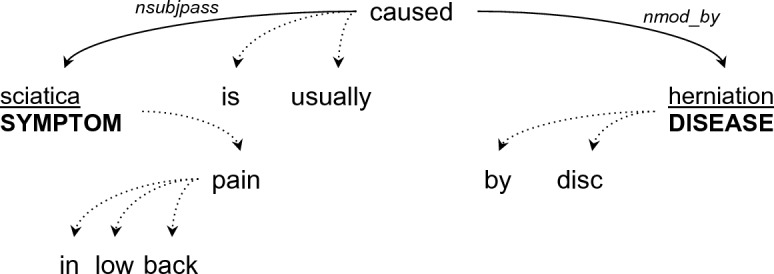
Dependency parse tree for the sentence “sciatica in low back pain is usually caused by disc herniation”. After applying the pattern generation algorithm we’ll obtain the pattern *[symptom placeholder] :*<*nsubjpass : lemm* = *cause :*>*nmod_by : [disease placeholder].*

The system derives a pattern from this input in the following way:

1. Obtain the shortest undirected syntactic dependency path *P* = *[A, n1, n2, …, B]* between *A* and *B* in *T***.**
2. If P includes only A and B (i.e. the two items are directly connected in T), find C which is the lowest mutual ancestor of A and B. If C exists, set P to be the path from A to C to B.
3. Derive a linearized pattern from P by walking through the items on the path, including the dependency edges between pairs of items and the lemmatized form of the words along the path. For example, for the sentence “[sciatica] in low back pain is usually caused by disc [herniation]” and the tree in Fig. 7, the generated path from “sciatica” to “herniation” is:

*[symptom placeholder] :* <*nsubjpass : lemm*=*cause :* >*nmod_by : [disease placeholder]*

The pattern will match sentences where a word with the lemma cause is connected by an outgoing *nsubjpass* edge to a word representing the symptom and by an outgoing *nmod_by* edge to a word representing an etiology. The matched words for symptom and etiology are automatically expanded by the SPIKE system to their linguistic phrase boundaries (e.g. if the word “herniation” is matched in the etiology position, it may be expanded to “disc herniation” or “lumbar disc herniation” depending on its surrounding context).

Note that superfluous elements not on the path such as “in low back pain”, “is usually” and “by disc” are dropped from the path so the resulting syntactic pattern will generalize better than a sequence pattern derived from the same sentence.

#### Pattern processing

Once a set of patterns is obtained, we use the SPIKE runtime to apply the patterns over PubMed. We obtain matching sentences and obtain sentences and pairs of symptom-etiology pairs in them which satisfy the syntactic restrictions. This results in a set of “Etiology Mentions”: sentences with marked etiology spans.

#### Grouping mentions into concepts

To group mentions into concepts we proceed with normalization and abbreviation detection:

- Normalization: we take from each raw mention its string fingerprint based on the steps below. We then unify all the mentions with the same fingerprint into a single concept.
  - We lowercase the name, remove punctuation, remove prefixes (a/an/the etc.), remove preceding/terminating spaces and turn multiple spaces into a single space
  - Split tokens on whitespace and remove duplicates
  - Sort the tokens to normalize word orders
  - If the resulting normalized name is greater than 6 chars, we run a Soundex algorithm (shorter strings remain as is).
- Abbreviation detection: we detect abbreviations using the Hearst Algorithm^30^ and replace them with their expanded form.
- Linking: we use the BioSyn entity linker^31^ to link the entities into the MEDIC disease ontology^32^. We take the top result of BioSyn (i.e. the entry name in MEDIC which is most likely to be associated with the entity) irrespective of its score, and use it as a concept ID. Since BioSyn always returns linking targets, the mentions will be given IDs even if a corresponding concept does not exist in MEDIC. However, since the IDs are only used for grouping it’s uncommon for this to pose a problem, as such entries will not typically be grouped with other entries, and will remain as singletons, maintaining their original (correct) name.

### Generating etiologies using ChatGPT

We used the May 12th, 2023 version of ChatGPT 4.0 (and in the ablations involving GPT 3.5, we used the version from the same date as GPT 3.5). ChatGPT has been used for Item Generation, Evidence Ranking and Evidence Selection, for Evidence Retrieval we used the SPIKE extractive search engine with a PubMed index which contains all PubMed abstracts published before February 2023. We experimented with different prompts and parameter options for the different stages. In all experiments, ChatGPT was run through the OpenAI API with temperature = 1, max_tokens = 4096 and top_p = 1. Below we specify the final prompts selected for each stage as well as some of the alternatives considered.

#### Item generation

To generate a list of items we used the prompt below, where <symptom> is replaced with one of our target symptoms (hiccups, jaundice or chest pain).

“Provide an exhaustive list of all possible <symptom> etiologies. Be succinct, list only names, each name in its own line. Include all known etiologies, even the most rare ones. write done when you finish listing all etiologies.”

To obtain the prompt we had 3 medical professionals spend 30 min trying out alternative prompts for symptoms they were familiar with (but not the ones tested) and send us the one that seemed best to them. We then compared the results of the 3 prompts on a target symptom (sciatica) and chose the best one. For each test symptom, we then ran the prompt 3 times and selected the run with the most results. Note that there is a difference in the number of results generated by GPT for the same symptom across runs (up to 17 fewer generated etiologies across symptoms in our results). Merging the results of multiple runs is non-trivial since GPT sometimes refers to the same etiology in slightly different names between runs. Still, if the issue of normalization is addressed, we believe this is an interesting direction for improving the recall of GPT. Also, ChatGPT can be asked to add more results to its initial result list. We experimented with this but found that it greatly increases hallucinations and variance.

#### Evidence retrieval

To retrieve evidence for a specific candidate etiology we used the dense retrieval options of the SPIKE engine based on the abstract embeddings described in Ravfogel et al. ^33^. In this case, SPIKE relies on a pre-populated embedding space which includes embeddings for all PubMed sentences. When given a query sentence, SPIKE retrieves the sentences which are semantically closest to it in vector space. The embeddings in Ravfogel et al. ^33^ are especially suited for cases where the search query contains an abstract term (e.g. “etiology”) and the matched sentences contain specific instances of the abstract term (e.g. specific diseases, etc.).

We used the query “an etiology for <symptom>” to find sentences which express a causal relation between an etiology and the symptom at hand. While similarity search is by design soft, we also added hard constraints requiring the results to contain the exact phrases for the etiology and symptom. We retrieved the top 50 matching sentences for each etiology.

Note that adding the hard constraints does mean we sometimes lose useful results (i.e. PubMed sentences where the symptom or etiology are mentioned in a different name than the one suggested by GPT). This is one of the causes of reduced recall by the “verified” GPT variant. However, as shown in the ablations in Table 8 below, without these hard constraints the number of etiologies with retrieved sentences increases (as expected, as the search is less stringent), but the retrieved sentences are less relevant, so after ranking and vetting the candidate evidence, the overall number of valid results for which evidence is found is decreased significantly.

**Table 8:** effects of requiring the appearance of the symptom and etiology (“hard constraints”) in the results of the dense query. The first number is the total number of etiologies for the symptom, the second is the number of etiologies for which we obtain candidate evidence through dense search, and the third is the number of etiologies for which the evidence also passed the final vetting stage.

	Dense retrieval + hard constraints	Dense retrieval
Chest pain	63, 56, 50	63, 63, 21
Hiccups	47, 36, 27	47, 47, 12
Jaundice	65, 53, 50	65, 65, 15

#### Evidence ranking

To rank the evidence we used the prompt:

“From the list of sentences below (one per line), which 3 sentences would be the most convincing ones to be used as evidence that <candidate_etiology> is an etiology for <symptom>?

When selecting the 3 sentences, prioritize sentences which explicitly state that the item belongs to the list over ones where this is only implied.

Only select sentences from the list below, do not add new sentences. If the list does not contain 3 sentences that could be used as supportive evidence, return fewer sentences, or 0 sentences (an empty list) if needed.

Format the results as a Python array, where each item is a dict with two items, the first item ("sent"), is the sentence itself. The second item ("reason") is a textual explanation of the reason why the sentence was selected.

Be succinct, reply with the Python list and nothing else.”

As evidence ranking is applied once per retrieved etiology it is potentially expensive to run at scale. We tested the effects of using GPT 3.5 turbo which is a magnitude of order cheaper than GPT 4.0. The results in Table 9 indicate a significant degradation when GPT 3.5 is used instead of GPT 4.0.

**Table 9:** effects of using GPT 3.5 for evidence ranking, instead of GPT 4. In each cell, we list the number of etiologies for which we could obtain evidence, out of the full set of etiologies which had candidate evidence sentences before ranking and verification.

	GPT 4.0	GPT 3.5
Chest pain	50/56	47/56
Hiccup	27/36	18/36
Jaundice	50/53	49/53

#### Evidence vetting

For evidence vetting, we used the following prompt.

“Can the following sentence be used as evidence that "<candidate etiology>" is an etiology for <symptom>? Be succinct, and reply with yes or no only.

The sentence is:

<evidence_sentence>”

As evidence vetting can potentially be expensive at scale, we tested the effects of using GPT 3.5 turbo which is a magnitude of order cheaper than GPT 4.0. The results are listed in Table 10. Compared to evidence ranking, where the use of GPT 4.0 appears critical, results of evidence vetting were less clearly impacted by the use of GPT 3.5 (2 symptoms showed better and slightly better results with GPT 4, while the third symptom showed slightly better results with GPT 3.5).

**Table 10:** effects of using GPT 3.5 for evidence vetting instead of GPT 4.0. In each cell, we list the number of etiologies for which we could obtain evidence, out of the full set of etiologies which had candidate evidence sentences before ranking and verification.

	GPT 4.0	GPT 3.5
Chest pain	50/56	52/56
Hiccup	27/36	22/36
Jaundice	50/53	49/53

## Discussion

In this study, we presented two novel methods to address the important medical problem of extracting symptom etiologies from the scientific literature. We show that generative models (ChatGPT) provide accurate results, especially when combined with the suggested verification pipeline, however, they struggle with retrieving the long tail of relevant etiologies. Patterns on the other hand have lower accuracy, but since they’re applied to an index of all Pubmed abstracts they provide much higher recall, compared both to GPT and the reference sources. Importantly, as shown in the Venn diagrams in Fig. 4 and the results in Table 1, both solutions turn out to be complementary and the best results are obtained by combining them. Below we discuss additional interesting aspects of this work.

### The problem of incomplete coverage

An interesting observation of this study is that even established manually curated clinical sources such as UpToDate or medical books are highly incomplete in their coverage of relevant symptom etiologies. Generative methods are also not ideal at covering the long tail of etiology data, as they struggle to encode rare facts in their limited parameter space^11^. Patterns applied over a large corpus (in this case the set of all PubMed abstracts) are still the most effective individual method at identifying the long tail of relevant information, but these also suffer from partial recall, not extracting the full set of etiologies identified by the other sources, and thus cannot fully replace them.

In the “Results”/“Qualitative analysis” section, we discuss the issues preventing the patterns from achieving higher recall, pointing out legal restrictions and data availability as significant issues, but also suggesting actionable steps which may improve recall with available data, namely applying the patterns to a larger part of the open scientific data (through the use of PMC-PubMed, in addition to PubMed abstracts), and addressing issues of missing patterns and findings organized in Tabular form.

The application of generative methods to this problem may also benefit from recent advancements in retrieval augmented language models^34^ and systematically applying such models to this problem may be a promising area for future research.

### Relevance to medical professionals

Louis Pasteur's famous quote: “In the fields of observation, chance favours only the prepared mind" means that the better prepared and more knowledgeable you are, the more you'll be able to take advantage of any chance opportunities or observations. Most of the landmark studies that have changed clinical medicine have been published in journals. Over the years, the number of active, peer-reviewed journals has expanded to approximately 28,000, collectively publishing more than 1.8 million articles every year^35^. In such an era, a ``prepared mind`` needs a tool to process such an amount of knowledge. We demonstrated the capacity to process vast volumes of medical literature and healthcare data in real time, thereby granting medical professionals access to the most current and comprehensive information available. This stands in stark contrast to manual searches, which often yield outdated or incomplete results, potentially culminating in misdiagnosis or delayed treatment decisions.

The ability to comprehend context and semantics within medical texts can aid clinicians in developing a reasonable differential diagnosis for the patient's symptoms. This aptitude allowed us to decode intricate medical jargon, identify subtle nuances in symptoms, and establish correlations with relevant etiologies, even when symptom causation was not the primary focus of the source material. In doing so, on one hand, we unearthed numerous etiologies that had not been previously documented in the reviews or the medical books, as potential causes for specific symptoms and on the other hand provided good precision in finding etiologies compared to the traditional tools used today while creating a big list of differential diagnoses for a set of symptoms.

Additionally, like most AI models, our tool can substantially alleviate the cognitive burden on medical professionals by automating the initial phases of symptom analysis and etiology identification. This automation empowers healthcare providers to redirect their expertise toward more critical tasks, such as patient interaction, treatment planning, and decision-making, ultimately bolstering the overall efficiency and effectiveness of healthcare delivery.

An important point at this time is that while AI-powered models undoubtedly offer numerous advantages in identifying symptom etiologies and enhancing healthcare delivery, it is essential to recognize that a significant portion of the population may harbour reservations regarding the integration of AI into their healthcare. According to a study conducted by the Pew Research Center, a noteworthy 60% of Americans express discomfort at the prospect of their healthcare provider relying on AI for their medical care^36^. Our research endeavours have demonstrated that a collaboration between AI developers and medical professionals along with a synergistic approach, merging generative models like ChatGPT with Syntactic patterns, yields both precise and extensive results. This amalgamation of AI and conventional methodologies could potentially serve as a crucial step in building trust within the population when it comes to embracing AI models within the medical domain. As the healthcare sector continues to explore the potential of AI, it becomes increasingly vital to leverage these tools effectively to not only enhance medical practice but also to foster confidence among patients and healthcare consumers.

In our research, we highlighted the profound relevance of AI in the realm of medical practice, particularly in the important task of identifying symptom etiologies. This cutting-edge technology presents a multitude of advantages when compared to the traditional methods commonly employed by physicians today.

## Data Availability

The datasets generated and/or analyzed during the current study are available from the corresponding author on reasonable request.
